# Exploring the Link between Oxidative Stress, Selenium Levels, and Obesity in Youth

**DOI:** 10.3390/ijms25137276

**Published:** 2024-07-02

**Authors:** Teofana Otilia Bizerea-Moga, Laura Pitulice, Otilia Bizerea-Spiridon, Tudor Voicu Moga

**Affiliations:** 1 Department XI of Pediatrics-1st Pediatric Discipline, Center for Research on Growth and Developmental Disorders in Children, ‘Victor Babeș’ University of Medicine and Pharmacy Timișoara, Eftimie Murgu Sq No 2, 300041 Timișoara, Romania; bizerea.teofana@umft.ro; 2 1st Pediatric Clinic, ‘Louis Țurcanu’ Children’s Clinical and Emergency Hospital, Iosif Nemoianu 2, 300011 Timișoara, Romania; 3 Department of Biology-Chemistry, West University of Timişoara, Pestallozi 16, 300115 Timişoara, Romania; otilia.bizerea@e-uvt.ro; 4 The Institute for Advanced Environmental Research (ICAM), Popa Şapcă 4C, 300054 Timişoara, Romania; 5 Department VII of Internal Medicine-Gastroenterology Discipline, Advanced Regional Research Center in Gastroenterology and Hepatology, ‘Victor Babeș’ University of Medicine and Pharmacy Timișoara, Eftimie Murgu Sq No 2, 300041 Timișoara, Romania; moga.tudor@umft.ro; 6 Gastroenterology and Hepatology Clinic, ‘Pius Brînzeu’ County Emergency Clinical Hospital, Liviu Rebreanu 156, 300723 Timișoara, Romania

**Keywords:** oxidative stress, biomarkers, selenium, obesity, children, adolescence

## Abstract

Obesity is a worldwide increasing concern. Although in adults this is easily estimated with the body mass index, in children, who are constantly growing and whose bodies are changing, the reference points to assess weight status are age and gender, and need corroboration with complementary data, making their quantification highly difficult. The present review explores the interaction spectrum of oxidative stress, selenium status, and obesity in children and adolescents. Any factor related to oxidative stress that triggers obesity and, conversely, obesity that induces oxidative stress are part of a vicious circle, a complex chain of mechanisms that derive from each other and reinforce each other with serious health consequences. Selenium and its compounds exhibit key antioxidant activity and also have a significant role in the nutritional evaluation of obese children. The balance of selenium intake, retention, and metabolism emerges as a vital aspect of health, reflecting the complex interactions between diet, oxidative stress, and obesity. Understanding whether selenium status is a contributor to or a consequence of obesity could inform nutritional interventions and public health strategies aimed at preventing and managing obesity from an early age.

## 1. Introduction

In recent decades, the global prevalence of obesity among children and adolescents has surged, posing significant public health challenges. This alarming trend not only predisposes young individuals to early-onset chronic diseases but also complicates the management of existing health conditions. Among the myriad of factors contributing to obesity, the interplay of oxidative stress and micronutrient status, particularly regarding selenium, has garnered increasing scientific interest. Oxidative stress, a biochemical imbalance between antioxidants and reactive oxygen species (ROS), is a pivotal factor in the pathogenesis of obesity and its related complications. Concurrently, selenium, an essential trace element with potent antioxidant properties, plays a crucial role in mitigating oxidative damage and maintaining metabolic health.

This narrative review aims to explore the intricate relationships between obesity, oxidative stress, and selenium status within this vulnerable population. By delving into the existing body of literature, through a comprehensive analysis of epidemiological, clinical, and mechanistic studies, it endeavors to provide insights into the complex interdependencies of oxidative stress, selenium status, and obesity, thereby informing future research directions and intervention strategies in pediatric populations.

This introduction sets the stage for a thorough examination of the existing research on the topic, highlighting the importance of understanding these relationships in addressing childhood and adolescent obesity.

## 2. Methodology of Literature Search

**Figure 1:**
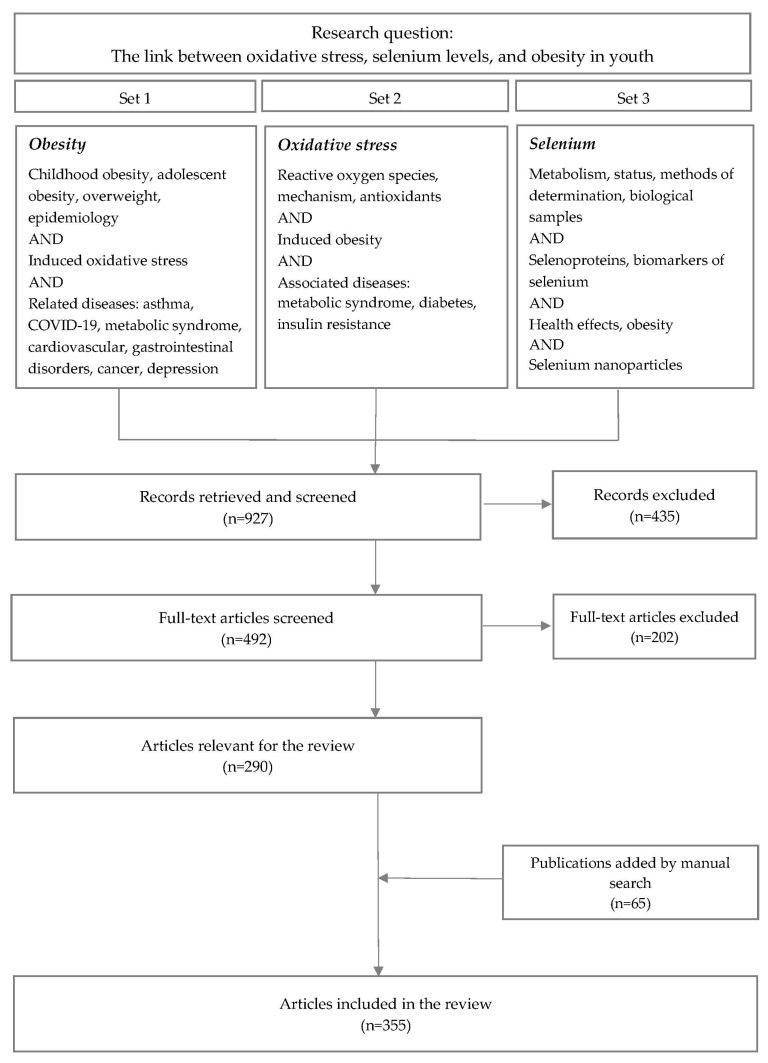
Literature search outline.

The methodology of this review was designed to capture the comprehensive landscape of research exploring the intersections of childhood obesity, oxidative stress, and selenium status. A search strategy was developed and applied across electronic bibliographic databases, including PubMed, Scopus, Web of Science, and the Cochrane Library. Iterative searches were performed with different combinations of terms and specific words or phrases (free text), as illustrated in Figure 1, in title or abstract headings (only) to generate a representative selection. Three distinct iterative queries were established: the first was focused on obesity prevalence in children and adolescents, and its relation with oxidative stress and associated diseases; the second was focused on oxidative stress mechanisms at the cellular level and the link with obesity and other disorders; and the third one focused on the role of selenium in the human body and its status in obese children. The search was limited to a 25-year period from 1 January 2000 to 31 December 2024. The time period covered by the search was set up with a future end date in order to also include accepted publications that are in the queue to be published.

The inclusion criteria allowed studies on children and adolescents with obesity or obesity-related disorders, presenting epi data or investigating the relationship between obesity and oxidative stress or selenium status, mechanisms, and biomarkers of interest. The publications included in this review fell under a range of designs including original research, systematic reviews, meta-analyses, clinical trials, cohort studies, and comprehensive reviews. Conversely, the exclusion criteria included short communications (editorial comments, letters, conference abstracts, or posters), case reports, series presenting diagnoses or treatments of obesity or obesity-associated diseases, and studies targeting different age groups (adults or elderly) or lacking focus on the topics of interest, without an available abstract as well as publications not written in English or German (the language restrictions were due to the availability of translation for data extraction).

The resultant citations were imported into a dedicated literature management software DistillerSR version 3.3 (DistillerSR Inc., Ottawa, ON, Canada, 2023) for review and adjudication.

The titles and abstracts of 927 studies were retrieved based on the search strategy, after the removal of duplicates. Records were evenly divided and reviewed by three of the authors at review Level 1. Level 2 resolved the uncertainties pertaining to the passing of articles to a full-text review and required the involvement of the fourth author. At Level 3, the articles were evenly split among all of the authors for full-text evaluation and subsequent data extraction. Data extraction focused on study design, participant characteristics, methodology used to assess obesity, oxidative stress, and selenium status, as well as the main findings related to these topics.

Of the 927 total records, 435 were deemed not applicable per the exclusion criteria. After Level 1 and Level 2 reviews, there remained 492 articles that were considered for further analysis (Figure 1). These 492 full-text articles were reviewed by the authors for relevance as described above, and a list of 290 articles was considered appropriate for the objective of the literature review.

The reference lists of some included publications were screened (backward citation chasing) as it provided an alternative way of accessing potentially relevant studies [1] for inclusion in this review. Hand-searching was performed as a useful adjunct. Thus, a total of 65 articles were manually added to the initial results.

This outline provides a clear and structured approach to conducting a thorough literature review, ensuring comprehensive coverage and critical analysis of the existing research on these interconnected topics. 

A range of topics of great interest for advancing knowledge in the field of oxidative stress and its biomarkers were assessed, such as the following: (1) the connection between selenium and the brain–gut–microbiota axis with consequences on both brain health and cognitive function, as well as on the composition of the intestinal microbiota; (2) reductive stress and its involvement in halting the proliferation of cancer cells; (3) synthetic and biogenic selenium nanoparticles that are more reactive, more compatible with the body, less toxic, and have a broader therapeutic window than other inorganic and organic forms of selenium; (4) hybrid bio-nanoarchitectures (bioactive molecules combined with selenium nanoparticles) with enhanced biological activity and reduced side effects, used in targeted and personalized therapies, medical imaging, and high-performance tissue engineering. 

Last but not least, the review tackles cutting-edge topics such as “multi-omics” technologies and biomarkers that offer significant opportunities in prevention, diagnosis, and treatment strategies for obesity and associated chronic diseases.

## 3. Discussion

### 3.1. Obesity in Children and Adolescents

#### 3.1.1. Obesity, an Epidemic Disease of the 21st Century—The Effects on the Health of Children and Adolescents

According to the World Health Organization (WHO), obesity is a “complex multifactorial medical condition”, characterized by excessive fat accumulation in the body, which can have a significant negative impact on both the physical and mental health of an individual. Obesity poses an increased risk for the development of numerous non-communicable diseases (NCDs). The WHO’s 2022 Regional European Obesity Report highlights obesity as a critical public health issue worldwide and a key factor in disability and mortality within the WHO European region. According to the report, being overweight and obese affects about one-third of children (29% of boys and 27% of girls) in the region, making it the fourth leading risk factor for NCDs, preceded only by arterial hypertension, unhealthy eating habits, and tobacco consumption. Research endorsed by the WHO indicates that being overweight, particularly obese, elevates the risk for 13 different types of cancer. Moreover, obesity is associated with an array of disabilities and plays a significant role in the heightened rates of morbidity and mortality linked to COVID-19 and other viral illnesses [2].

Although the prevalence of being overweight or obese worldwide varies significantly due to various factors such as geographic region, country, race, ethnic group, gender, age group, culture, diet type, socioeconomic conditions, and genetic factors, an alarming upward trend has been consistently observed since the last decade of the previous century until present. The worrisome increase in obesity rates among children and young people is a major concern for all organizations involved in public health monitoring since this alarming trend can contribute to the perpetuation of the global obesity epidemic with serious consequences for the future health and well-being of these population groups.

The recent COVID-19 pandemic affected society as a whole, but its impact has been more pronounced among children and adolescents. Several lifestyle changes, such as social isolation, reduced outdoor time, closure of educational institutions with a shift to online learning, prolonged screen time encouraging sedentary behavior, and affected eating habits, as well as the duration of rest and recovery, have contributed to the increased prevalence of obesity and other related morbidities [3,4].

Childhood and adolescent obesity can significantly impact health, leading to disruptions in the proper functioning of various organs and systems in the body (cardiovascular, endocrine, reproductive, digestive, immune, respiratory, skeletal, muscular, excretory, nervous, etc.) [5]. The consequences of these disorders are severe and can increase the risk of developing short-term or long-term conditions such as the following:

1. High blood pressure and imbalances in lipid metabolism resulting in dyslipidemia, heart disease, and stroke [6,7,8,9,10,11,12,13];
2. Hormonal imbalances, insulin resistance, hyperglycemia, metabolic syndrome (MetS), and type 2 diabetes [14,15,16,17,18];
3. Impaired reproductive function and polycystic ovary syndrome [19,20,21,22,23];
4. Non-alcoholic fatty liver disease, gastroesophageal reflux disease [24,25,26,27,28,29];
5. Weakened immunity and increased susceptibility to bacterial infections and viral diseases [30,31,32,33,34,35];
6. Asthma and other respiratory conditions [36,37,38,39,40,41,42,43];
7. Obstructive sleep apnea and other sleep disorders [44,45,46,47,48,49,50,51];
8. Joint pain, osteoarthritis, and other neuromusculoskeletal impairments [52,53,54,55,56,57];
9. Chronic kidney disease with an increased risk of developing renal failure [58,59,60,61,62,63,64,65,66];
10. Activation of the sympathetic nervous system regulating the stress response, various mental health conditions, etc. [67,68,69,70,71].

Childhood and adolescent excessive weight and obesity can also lead to various psychosocial complications related to social isolation, low self-esteem, increased stress levels, anxiety, depression, and attention-deficit/hyperactivity disorder (ADHD) [72,73,74,75,76]. Moreover, studies suggest that childhood and adolescent obesity, along with other factors (genetic predisposition, lifestyle, pollution, exposure to carcinogenic substances and radiation, some viral/bacterial infections, etc.), may be risk factors for developing certain forms of cancer in adulthood. The connection between increased body mass index (BMI) and malignant diseases is not fully understood, but several physiological mechanisms associated with obesity (chronic inflammation, endocrine dysfunction, insulin resistance, etc.) can pose risks for developing adult cancers (leukemia, Hodgkin’s disease, colorectal cancer, breast cancer, etc.) [7,77,78,79,80,81,82]. Indeed, obesity can negatively influence the health and well-being of both children and their families, generating physical health issues and emotional and psychological problems [83,84]. 

Obesity affects infants (0–1 years) and toddlers (1–3 years), preschoolers (3–5 years), school-age children (5–18 years), and adolescents (10–19 years), respectively. The 2022 WHO report indicates that excess weight (including obesity) affects 4.4 million infants, toddlers, and preschoolers, accounting for 7.9% of all children under the age of five. Additionally, almost one-third of school-age children are overweight or obese, with the prevalence temporarily decreasing to one-fourth among adolescents. In adults, the situation is even more serious, with rates rising to almost 60% of the population [2]. 

#### 3.1.2. Definitions

Childhood obesity is measured using the body mass index, a weight-to-height ratio that is calculated using the formula BMI = weight (kg)/height (m)^2^ [85].

If a child’s BMI exceeds a certain age and gender-specific threshold, they are considered overweight or obese. While various definitions exist for childhood obesity, none are universally accepted, complicating its study. Additionally, defining obesity has evolved to emphasize excess body fat, with BMI as the key metric. However, applying the BMI to children and adolescents is complicated due to age-related changes and gender differences. The approach involves comparing a child’s BMI against a reference population of healthy children and adolescents. Corresponding to age and gender, the percentiles of the BMI used to classify weight status are listed in Table 1.

Based on the BMI reference standards from the 2000 Centers for Disease Control (CDC) Child Growth Charts, childhood overweight is defined by a BMI at or above the 85th percentile and below the 95th percentile for age and gender, while obesity is defined by a BMI at or above the 95th percentile, in children aged two years and older [86,87]. For younger children, the CDC suggests using the WHO’s charts, which are specific to age and gender for weight-for-length measurements, instead of using the BMI [88]. 

It should be noted that although the BMI is frequently used to evaluate weight status, this parameter is estimative and does not provide a complete picture of a person’s body composition. It does not differentiate between fat and muscle mass and cannot assess fat distribution. The BMI is particularly estimative in the case of children and adolescents whose body composition changes during growth. For this reason, the BMI can serve as an initial indicator of the assessment of weight status in children and adolescents, but it is essential to complement it with other parameters, such as body composition, a ratio of fat to muscle mass, and the estimation of fat distribution, using additional non-invasive techniques like measuring skinfold thickness or bioimpedance analysis. Since all these methods for evaluating weight status have limitations and are influenced by various individual factors, (e.g., a person’s hydration level) the results must be corroborated with information regarding the level of physical activity and the medical history of each individual [5]. 

### 3.2. Oxidative Stress and Obesity, a Vicious Circle with Serious Consequences

Oxidative stress (OS) and obesity potentiate each other effects, impacting health and potentially leading to severe chronic conditions [89,90,91,92].

Oxidative stress occurs when the production of free radicals in the body (reactive oxygen or nitrogen species) exceeds the body’s capacity to neutralize them through antioxidants. Various factors such as exposure to radiation, pollution, including food pollution, an inadequate diet, smoking, a sedentary lifestyle, excessive stress, and others contribute to its occurrence. The excess of free radicals affects cells and damages the cellular signaling pathway [93,94,95]. Hence, OS can contribute to premature aging and the development of severe conditions, such as chronic fatigue syndrome, ADHD, cardiovascular diseases (hypertension, atherosclerosis, and myocardial infarction), endocrine diseases (insulin resistance and diabetes), MetS (a complex of metabolic, cardiovascular, and endocrine disorders), neurological diseases (Alzheimer’s and Parkinson’s), autoimmune inflammatory diseases (rheumatoid arthritis and systemic lupus erythematosus), and certain types of cancer [96,97].

Obesity represents a complex pathology of a multifactorial nature, resulting from the interaction of various factors (genetic, metabolic, behavioral, and environmental). It is a chronic condition with risk factors contributing to its development and progression, including lifestyle, diet, physical activity level, genetic predisposition, social and economic environment, prolonged stress, etc. [98,99,100]. It is characterized by the excessive accumulation of body fat and is the result of an imbalance between caloric intake (calorie consumption through diet) and caloric expenditure by the body (calories burned through basal metabolism and physical activity) [101,102]. Obesity constitutes a major risk factor for several serious conditions such as insulin resistance, hyperglycemia, (MetS), type 2 diabetes, hypertension, cardiovascular diseases, stroke, weakened immunity, bronchial asthma, non-alcoholic fatty liver disease, infertility, and certain types of cancer [7,23,24,25,26,27,30,31,32,33,34,35,36,37,38,39,40,41,42,43,59,77,78,79,80,81,82,100,102,103,104,105,106,107,108,109,110,111,112].

The bidirectional connectivity between obesity and oxidative stress is complex and often harmful. Although research has highlighted the reciprocal potentiation effect of oxidative stress and obesity, the joint action mechanism and whether oxidative stress is a triggering factor or a result of obesity, are still not fully understood. In fact, this is a vicious circle with an unknown cause–effect chain [90,113,114].

On the one hand, numerous studies focus on the influence of OS and chronic inflammation at the cellular level on the MetS and its components, including obesity. However, as noted by Masenga and colleagues in a recent review on this topic, few of these studies emphasize the mechanisms by which OS contributes to the onset and development of MetS/obesity. Additionally, there are even fewer studies focusing on the use of oxidative stress biomarkers in diagnosing and estimating the severity of these conditions [115]. 

Oxidative stress can activate the deposition of adipose tissue in the body by stimulating the proliferation of preadipocytes, disrupting their differentiation into mature adipocytes, and increasing the size of mature adipose cells through inflammation and mitochondrial dysfunction. By combining these mechanisms, oxidative stress can become an important factor that triggers and develops obesity [114,116,117]. 

Moreover, OS can disturb the carbohydrate and lipid metabolism of the cell, leading to the development of insulin resistance and fat accumulation in tissues. The disruption of the carbohydrate mechanism is due to OS interfering with insulin signaling pathways by modifying the structural insulin receptors on the cell surface and reducing their ability to bind to the hormone. Additionally, OS can disrupt the energy-generating mitochondrial function required for cells to respond to insulin signaling, uptake it from the blood, and regulate metabolism. Cell resistance to insulin uptake leads to an increase in circulating blood glucose levels (hyperglycemia) and higher insulin secretion for a compensatory effect. As insulin resistance worsens, it can progress to type 2 diabetes. Hyperglycemia can contribute to and exacerbate the inflammatory process by stimulating the production of pro-inflammatory cytokines [118,119,120,121].

Insulin regulates the lipid metabolism of the cell by signaling the uptake of glucose by adipose cells and storing it in the form of triglycerides. Therefore, by manipulating insulin and its signaling pathways, OS can contribute to excessive fat storage in cells, leading to obesity. Furthermore, OS can contribute to the excessive accumulation of lipids in the blood and can affect the walls of blood vessels by increasing vascular permeability, facilitating the infiltration of lipids from the blood into tissues. This is another way to achieve excessive fat accumulation in tissues and organs, and thus cause obesity. All these processes triggered by OS are interconnected, and mutually potentiate and exacerbate the metabolic imbalance and pathologies caused by it, including obesity [122,123,124,125,126]. 

In addition, OS, through the excess of oxidant species, can activate specific transcription factors, sensitive to redox processes, which, in turn, can trigger chain reactions releasing pro-inflammatory cytokines. These protein molecules can activate intracellular inflammatory signaling pathways in white adipose tissue and initiate a chronic inflammatory response associated with obesity. At the same time, they increase the production of reactive oxygen species in adipose cells, thereby contributing to the occurrence and maintenance of OS. Therefore, inflammation associated with obesity exacerbates OS, which, in turn, sustains or even amplifies inflammation [90,113]. 

Oxidative stress can affect the structure and composition of the intestinal microbiome, the balance between probiotic and pathogenic bacteria in favor of harmful ones, causing microbial dysbiosis with negative consequences on digestion and nutrient absorption. These changes can impact weight and metabolism. Additionally, OS, through the excessive production of ROS and RNS, can cause inflammation and damage to the intestinal mucosa. A compromised intestinal barrier can affect the absorption of nutrients and allow the passage of undigested bacteria and particles into the blood, triggering a generalized inflammatory reaction in the body. There are studies showing that genetic factors, environmental factors, and lifestyle contribute to the occurrence of a redox imbalance at the cellular level correlated with the alteration of the intestinal microbial flora, which plays a crucial role in regulating metabolic and inflammatory processes in the body [89,127]. 

Oxidative stress also influences the production and regulation of hormonal homeostasis in the body by affecting the sensitivity of hormone receptors and amplifying hormone secretion. As a result, OS can lead to an increase in the level of stress hormones (cortisol), hunger hormones (ghrelin), as well as the satiety hormone (leptin), which can influence appetite and eating behaviors, with serious consequences regarding the consumption of calorie-rich and carbohydrate-rich foods. The mechanism by which OS promotes obesity through leptin is complex. Since leptin is mainly produced by adipose tissue, a common metabolic change observed in obesity is the increase in leptin concentration in blood. In obesity, adipocyte hyperplasia and increased leptin levels affect the leptin signaling receptor in the brain, developing so-called leptin resistance. Signals of satiety sent by leptin are not correctly interpreted by the brain, leading to overeating and continuous fat accumulation. On the other hand, leptin plays a crucial role in obesity-induced OS by activating nicotinamide adenine dinucleotide phosphate (NADPH) oxidase and increasing the production of reactive species such as hydrogen peroxide and hydroxyl radicals, thereby amplifying OS in the body by stimulating the production of ROS and reducing the activity of antioxidants [116,128,129].

On the other hand, the existing body of literature is relatively poor in terms of references to the activation and augmentation mechanisms of OS due to obesity and other components of MetS [116,117,128,129]. Despite the recent and controversial nature of approaches, researchers agree on the complexity of the mechanisms by which obesity can induce OS. In this regard, several mechanisms have been recognized through which excessive fat deposits, chronic inflammation, and metabolic imbalances associated with obesity can contribute to an increased OS in the body.

It has been observed that obesity is associated with a chronic systemic inflammatory process. Adipocytes and other cells of adipose tissue are involved in the production of pro-inflammatory cytokines and adipocytic hormones that can trigger chronic inflammatory conditions in the body. At the same time, adipose tissue allows the excessive accumulation of macrophages, which can also release a series of inflammation mediators, amplifying the process. Established chronic inflammation can contribute to the onset and development of insulin resistance and other metabolic changes. A vicious circle is created because insulin resistance, associated with obesity, causes an increase in blood sugar levels, which, in turn, can contribute to increased inflammation. In addition to this, systemic inflammation can contribute to disrupting the redox balance in adipose tissue cells. NADPH oxidase is an enzymatic complex that catalyzes the production of ROS, including superoxide, as part of the inflammatory response, in cells of the immune system and adipose tissue. Therefore, hypertrophy of adipose tissue induces chronic inflammation in the body, disrupting mitochondrial function, activating NADPH oxidase, increasing ROS concentration, and hence OS alongside the generation of superoxide through the action of NADPH oxidase, glycerol aldehyde auto-oxidation, oxidative phosphorylation in mitochondria, activation of protein kinase C, and metabolic pathways of polyol and hexosamine [116,117]. For example, in the process of oxidative phosphorylation in mitochondria through which cells produce energy, ROS such as superoxide and hydrogen peroxide can be formed. In case of excessive body fat accumulation, cellular energy metabolism is affected due to the disruption of oxidative phosphorylation, leading to an excess of ROS, and thus, OS is established. The oxidation of fatty acids in mitochondria (beta-oxidation) provides the energy needed for various cellular activities and vital functions. On the other hand, lipid peroxidation, the degradation of lipids (including fatty acids) in the presence of free radicals and other ROS, plays an important role in cell signaling, protection against infections, and regulation of the inflammatory response. In obese subjects, the oxidation of fatty acids and lipid peroxidation are deregulated. Therefore, a higher amount of fat stored in individual adipocytes in obesity induces the formation of ROS and inflammation. This is another way obesity exacerbates OS and supports the vicious circle of interrelated mechanisms obesity ↔ oxidative stress, and oxidative stress ↔ obesity [128].

Oxidative stress and inflammation associated with obesity can also affect cellular DNA. Highly ROS can modify the structure and sequence of DNA by breaking the chain and/or damaging nucleotide bases. In turn, the inflammatory process that occurs in obesity can inhibit DNA repair mechanisms. Together, these two factors can lead to genetic mutations and the development of extremely serious pathologies [129]. 

MicroRNAs (miRNAs) play an intricate role in the relationship between childhood obesity, MetS, and OS. miRNAs serve a dual function: they can be influenced by ROS and conversely, affect the body’s antioxidant defenses. Oxidative modifications to miRNAs can lead to incorrect mRNA targeting, impacting processes like cardiac hypertrophy and cell apoptosis. Furthermore, miRNAs regulate mitochondrial functions and adipogenesis, affecting mitochondrial health under conditions like hyperlipidemia and hyperglycemia. Various miRNAs have been shown to regulate proteins associated with inflammation and glucose metabolism, impacting endothelial function and the progression of atherosclerosis. Additionally, miRNAs can disrupt metabolic pathways leading to endoplasmic reticulum stress, which is linked to hyperlipoproteinemia common in MetS. Specific obesity-related miRNAs have been identified to target mechanisms producing ROS, highlighting their potential in moderating oxidative damage, and suggesting a role in preventing metabolic diseases in obese patients by reducing OS. miRNAs show potential as central regulators of antioxidant enzyme expression and activity in obesity. By influencing various signaling molecules and enzymes, miRNAs can directly and indirectly affect oxidative metabolism, suggesting their importance in the pathophysiology of metabolic disorders. Therefore, they could be targeted therapeutically to manage obesity-related conditions by modulating their effect on OS and cell damage [130,131,132,133,134,135,136,137,138,139]. 

In conclusion, each factor related to OS that causes obesity and, conversely, obesity that induces OS is part of a vicious circle, a complex chain of mechanisms that derive from each other and reinforce each other, without a tendency toward balance and with effects that are most harmful. Oxidative stress can affect cell receptivity by creating insulin resistance, disrupting the normal function of mitochondria, inducing metabolic dysfunction at the cellular level, triggering an inflammatory state in the body by increasing the production of proinflammatory cytokines, and affecting the balance of the microbiota. All these factors can lead to fat accumulation and the development of obesity. On the other hand, the excessive accumulation of fat characterizing obesity, as well as the chronic inflammation and metabolic imbalance associated with it, can increase the production of free radicals and decrease the concentration of antioxidants, leading to an increase in OS levels in the body.

### 3.3. Mechanisms by Which Selenium and Its Compounds Affect Health

**Figure 2:**
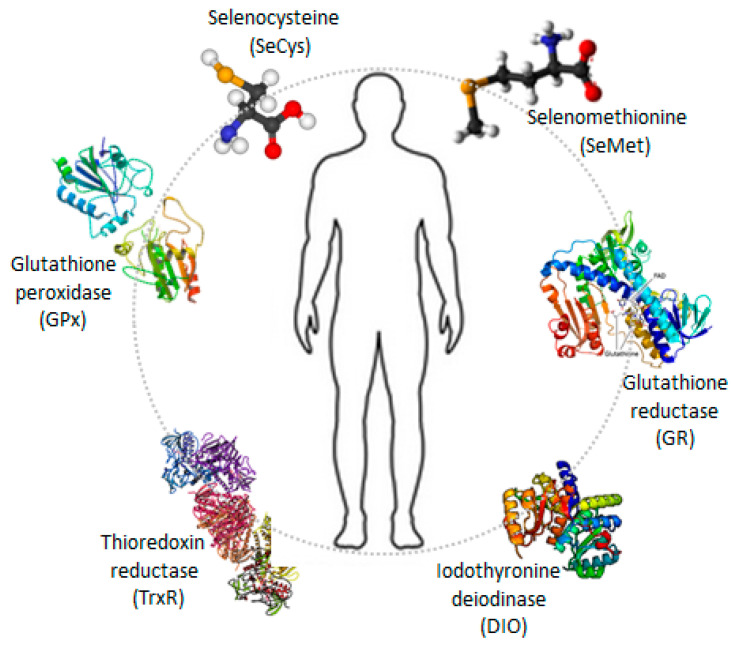
Selenium organic compounds present in the human body.

Selenium compounds enter the body from food and supplements through ingestion and are absorbed in the digestive tract, but they can also be taken up from the environment through the respiratory system or through direct contact with the skin. Selenium species are retained in the body although more in the organic forms (Figure 2) than in the inorganic forms. Organic selenium is recognized as an amino acid being non-specifically metabolized and incorporated into the structure of proteins. This selenium is only released when proteins break down, not when the body needs it, leading to an accumulation of selenium in the tissues [140].

Although present in extremely small quantities in the body, selenium is involved in a series of key biochemical processes. Through selenium-containing proteins such as glutathione peroxidase (GPx), thioredoxin reductase (TrxR), selenoprotein R (SelR or SelenoR), selenoprotein O (SelO or SelenoO), and other selenoprotein enzymes essential for cellular redox regulation, selenium facilitates the neutralization of excess free radicals, contributing to antioxidant defense, balancing the oxidant/antioxidant ratio, maintaining redox homeostasis, and protecting cells against OS [96,141,142,143,144].

Replacing sulfur atoms with selenium atoms, or thiol groups with selenol groups in the amino acids that compose selenoproteins, provides selenium compounds with a major catalytic advantage in terms of faster kinetics in oxidation reactions and significantly better reversibility under biological conditions. Thus, one of the main reasons selenium in the form of selenocysteine is used in protein construction is because it exhibits “oxygen tolerance”, meaning it resists permanent oxidation. This is due to a dual quality. First, selenium exhibits a strong nucleophilic character, allowing it to attack electrophilic agents, acting as a powerful reducer of free radicals that neutralize them and thereby contributing to cell protection against OS. Second, the selenium–carbon bond is weaker, more labile (easier to cleave), and breaks more easily than the sulfur–carbon bond, making selenium oxidation reactions more pronouncedly reversible. Once selenium has been oxidized in a reaction, it can relatively easily return to the reduced state by modifying the reaction conditions. This reversibility is crucial in catalytic processes and maintains the function of selenoproteins within optimal parameters [145,146]. Due to the catalytic role in redox reactions and the antioxidant activities of selenoproteins, selenium helps regulate and maintain essential cellular processes such as signal transduction, proliferation, cell transformation, aging, apoptosis, and ferroptosis (a distinct form of iron-dependent programmed cell death) [147,148,149,150,151,152].

Numerous studies have concluded that OS and the associated free radicals cause a series of damages at the cellular level. Thus, some functional groups, such as thiol groups (-SH), alcohol groups (-OH), and amino groups (-NH_2_) from constituent amino acids of proteins (cysteine, serine, and threonine, as well as lysine and arginine) can be oxidized by ROS and RNS. Additionally, unsaturated double or triple bonds in the side chains of amino acids in proteins may undergo oxidations, with dramatic consequences on the structure, stability, and function of cellular proteins. Moreover, the oxidation of these protein components can lead to the formation of stable and highly reactive products such as protein hydroperoxides, which can initiate polymerization reactions in compounds with unsaturated chemical bonds, as well as electrophilic molecules that can interact with nucleophilic side chains of proteins, leading to structural and functional damage. Free radicals can significantly impact cells due to the oxidative degradation of polyunsaturated fatty acids in the cell membrane. Membrane lipid peroxidation can affect the structure, fluidity, permeability, and biophysical properties of cellular membranes. These changes disrupt the functionality of cellular membranes, affecting signal transmission, cellular communication processes, selective substance transport, maintenance of the specific intracellular environment, and the preservation of cellular homeostasis. Oxidative stress can also affect the genetic material of a cell by modifying the nitrogenous bases of DNA, single and double DNA strand breaks, translocations, deletions, or chromosomal inversions, as well as the alteration of gene expression. All these effects can lead to the occurrence of genetic mutations and the development of serious diseases [153]. Considering all these negative effects of OS at the cellular level, another important function of selenium and its compounds relates to the biosynthesis and maintenance of DNA and RNA, as well as the regulation of gene expression. For example, TrxR is involved in DNA repair processes and plays an important role in regulating gene expression through redox control exerted on the structure and activity of transcription factors. Additionally, recent studies on animals have demonstrated that selenium, in the form of nanoparticles, protects proteins and DNA from the negative effects of radiation, acting as a good genetic and radioprotective agent [143,154,155,156]. Through its compounds, such as GPx, TrxR, as well as the three deiodinases, selenium contributes to the synthesis of thyroid hormones and their conversion from an inactive form to an active form, thereby regulating basal metabolism in all tissues of the body. These selenoproteins play an important role in hormonal metabolism and are involved in combating free radicals generated during the production of thyroid hormones [148,157,158]. Two other selenium compounds in the body, selenoprotein-S (SelS or SelenoS) and selenoprotein-P (SelP or SelenoP), present mainly in the blood plasma but also in other tissues and make major contributions to supporting the immune system function, regulating the inflammatory process, protecting cells against damage caused by OS, ensuring organism homeostasis, and even wound healing [143,159]. Regarding the antioxidant activity of selenium and its compounds, their involvement in cardiovascular protection can also be mentioned through their protective effect on blood vessels against OS, chronic inflammation, endothelial dysfunction, vascular calcification, and vascular cell apoptosis. Studies have shown that the disruption of antioxidant defense provided by GPx, as well as ferroptosis, closely linked to the activity of this selenoprotein enzyme, represents critical elements in the pathogenesis of myocardial injury and other cardiovascular pathologies [160,161,162].

It is widely recognized that there is an interaction between adiposity, OS, and the risk of developing cardiometabolic diseases, including cardiovascular diseases, MetS, and type 2 diabetes. Thus, it is known that excess adipose tissue, especially in the abdominal area, is associated with a systemic inflammatory response, which, in turn, is linked to an increased risk of OS. On the other hand, the increased generation of ROS in excess adipose tissue is implicated in the development and progression of cardiovascular diseases and type 2 diabetes. Therefore, in addition to the direct involvement of selenium and its compounds in cardiovascular protection through their antioxidant action and combating of OS, these species can also indirectly influence the pathogenesis of cardiometabolic diseases by regulating metabolism, preventing obesity development, and reducing inflammation associated with it [91].

As a high oxygen consumer, nerve cells inherently generate a significant number of free radicals during cellular metabolism, contributing to an increase in OS with negative effects on the health of the nervous system. Through their involvement in cellular redox processes, GPx and other selenoproteins, such as selenoprotein-P, TrxR, iodothyronine deiodinase, etc., directly influence neuronal activity, including neurotransmission, and contribute to the protection of nerve cells and the proper functioning of the central nervous system. Especially, selenoprotein-P is essential for the proper functioning of the brain due to its role in selenium transport in the body, including towards the brain [163,164,165].

The complex communication and regulation system between the central nervous system (brain), enteric nervous system (nervous tissue in the digestive tract), and intestinal microbiota (all bacteria and other microorganisms in the intestine) is known as the brain–gut–microbiota axis. This axis contributes to regulating digestive functions, maintaining mental health, and ensuring the proper functioning of the immune system. The role of selenium and selenoproteins in this relationship is significant. Thus, their antioxidant and anti-inflammatory activities can be reflected both in the brain and intestines, protecting their interaction with the microbiota. Additionally, selenium can balance the microbial flora, preventing health damages associated with the disturbance of the composition and normal functioning of the intestinal microbiome (dysbiosis). In fact, the relationship between selenium status and the intestinal microbiota is bidirectional. On one hand, selenium is a modulator of the composition and function of the intestinal flora because the intestinal microbiota uses this trace element for the synthesis of its own selenoproteins. On the other hand, the microbiota is an environment that affects selenium status as it can favor the biotransformation of its compounds and generate metabolites capable of interacting with selenium or selenoprotein enzymes [166,167]. 

The mechanisms by which an adequate selenium level in the body helps in fighting against various forms of cancer are not fully clarified, although many studies have been conducted in this regard. Generally, the anticancer action of selenium is due to the antioxidant properties of selenoproteins, given that ROS and, especially, free radicals can contribute to cancer development through damage to DNA and other cellular components. It has also been found that selenium can modulate cell proliferation and programmed cell death, contributing to the control of cell division and slowing down the uncontrolled cell growth characteristic of tumorigenesis, inducing apoptosis in cancer cells. In addition, in the cancer development process, the growth and survival of cancer cells generate important changes in energetic metabolism and immune response at the cellular level. By modulating cellular energy metabolism and enhancing the immune system, selenium and its compounds are particularly important allies in the fight against cancer. A recent approach regarding how selenium and its metabolites are involved in stopping the proliferation of cancer cells appeals to the notion of reductive stress. Unlike OS, the mechanisms that induce reductive stress, the responsible reducing species, and how cancer cells respond to reductive stress are less studied. Nevertheless, it can be considered that selenium and its compounds play a dual role in combating cancer by neutralizing oxidative stress simultaneously while inducing reductive stress. The involvement of oxidative stress in cancer development refers to the action of ROS that can damage cells and contribute to tumorigenesis. Selenium and its compounds have an indirect anticancer action by neutralizing the excess of ROS and counteracting OS. On the other hand, reductive stress refers to the accumulation of reducing species in normal or tumor cells. It has been observed that the formation of extremely reactive and reducing metabolites, such as hydrogen selenide (H_2_Se), could have a direct anticancer action by stopping the cellular processes of growth and survival of cancer cells and inducing their apoptosis. It can also be assumed that a subtle balance and a selective and/or cumulative effect of selenium oxidant and reducing species within tumor cells can constitute an efficient mechanism of its anticancer action [168,169]. 

Finally, selenium plays a very important role in fertility and reproductive function in both men and women by being involved in the production and proper functioning of sex hormones, as well as in the protection of reproductive cells against OS. Numerous aspects related to aging, involving OS, imbalances in the antioxidant defense system, and the decreased ability of the body to neutralize free radicals, vascular diseases, especially atherosclerosis and hypertension, diabetes, especially type 2, infection of reproductive glands, and obesity, more common among the elderly, play an important role in age-related reproductive dysfunction [170].

Therefore, selenium is essential for the health and proper functioning of living organisms. Both deficiency and excess of this trace element can be very harmful to health because the element contributes to maintaining immune, endocrine, metabolic, and cellular homeostasis. The consequences of insufficient selenium intake are devastating, as its compounds protect against severe conditions associated with low selenium levels in the plasma [141,171,172,173,174,175,176]. As seen, a low selenium status in the body can be associated with an increased risk of mortality from various causes, a significant decrease in protection against OS, deficient immune function, disruption of inflammatory processes, cognitive decline, and thyroid dysfunction [177]. 

Numerous experimental and observational studies, reviews, and meta-analyses support the idea that selenium deficiency can contribute to the development or worsening of serious diseases and conditions such as (1) cardiovascular diseases (Keshan endemic disease, atherosclerosis, coronary artery disease, heart failure, and myocardial infarction) [160,178,179,180], (2) neurological and neurodegenerative disorders (autism, epilepsy, cerebral palsy, peripheral neuropathy, amyotrophic lateral sclerosis, Parkinson’s disease, and Alzheimer’s disease) [181,182,183,184,185,186,187,188,189], (3) viral/bacterial infections and acute respiratory diseases (hepatitis, HIV/AIDS, COVID-19, acute respiratory distress syndrome, pneumonia, skin infections, streptococcal infections, and tuberculosis) [190,191,192,193,194,195,196], and (4) various types of cancer [168,197].

Synthetic selenium nanoparticles, especially biogenic ones, exhibit a higher level of absorption and distribution in organisms, enhanced compatibility with human organs and tissues, increased metabolism, consequently significantly reduced toxicity, and a much broader therapeutic window. They also show superior reactivity compared to other inorganic and organic forms of selenium. Moreover, they excel in terms of bio-disponibility, bioactivity, controlled transport, and balanced release of selenium compounds (selenoproteins, selenoenzymes, etc.). Additionally, selenium nanoparticles can be employed as a carrier medium for various therapeutic substances, enhancing the healing effects of medications [155,174,198,199,200,201,202,203]. For these reasons, there has been a particular focus on studying their effectiveness, especially regarding antioxidant activity, which has implications for stabilizing the immune system and activating the body’s defense response. The efficacy of these selenium nanospecies is also being monitored in controlling inflammation, combating bacterial and viral pathogens, stimulating the body’s immune response, and inhibiting the proliferation of cancer cells [199,204]. The use of selenium nanoparticle therapies is considered for conditions where inflammation and OS represent major pathogenic pathways, including arthritis, inflammatory bowel diseases, fungal, bacterial, viral, and parasitic infections, obesity, MetS, diabetes, nephropathy, cardiovascular diseases, autoimmune diseases, neurodegenerative diseases (Alzheimer’s, Parkinson’s, epilepsy, ischemic stroke, etc.), polycystic ovary syndrome and reproductive diseases, drug resistance, and various types of cancer [149,198,199,200,202,203,204,205,206,207,208,209,210,211,212,213,214,215,216,217,218,219,220]. Due to these advantages, selenium nanoparticles are envisioned as an alternative to current forms of selenium administration, including supplements, although the clinical application of results obtained in preclinical and laboratory research faces some obstacles. A recent breakthrough involves the development of bioactive materials with multifunctional attributes based on bio-nanoarchitectures. Hybrid bio–nano systems with selenium can be obtained by combining bioactive molecules (enzymes, proteins, and DNA) with selenium nanoparticles or their compounds. The integration of nano and bio counterparts with synergistic actions in selenium-based nanohybrid systems has paved the way for various applications in multifunctional biomedicine, including targeted and personalized therapies with enhanced biological activity and reduced side effects; advanced sensors and probes for diagnosing and monitoring medical conditions; improved medical imaging providing clearer and more detailed images of tissues and biological structures; and efficient tissue engineering [221,222].

### 3.4. Biomarkers for Assessing Selenium Status in the Body

The existing literature documents a robust association between OS and obesity, underscoring their substantial effect on health outcomes. Oxidative stress may facilitate the onset of obesity, while obesity itself can exacerbate OS within an organism. A comprehensive elucidation of the underlying mechanisms governing this reciprocal relationship is imperative for the formulation of more efficacious therapeutic strategies aimed at the management of obesity and its related health complications.

Obesity itself constitutes a condition of elevated OS, attributed to the diminution of the body’s antioxidant defenses and, consequently, an overproduction of ROS. These observations have facilitated the identification of OS biomarkers associated with obesity, which can be employed to assess the condition’s status [95,223]: 

(1) Vitamin C (ascorbic acid) and vitamin E (α-tocopherol). These represent exogenous antioxidants that combat OS. An inadequate diet often associated with obesity and characterized by high fat and carbohydrate content but low in whole grains, fruits, and vegetables, can result in diminished levels of these vitamins in the body. Consequently, reduced serum concentrations of these biomarkers indicate a compromised defensive capacity of the organism against OS [95,96,224,225].

(2) Glutathione peroxidase (GPx). It is a family of endogenous selenium-containing antioxidant isoenzymes. They protect cells against lipid peroxidation in cell membranes by removing free peroxide from the cell. Together with glutathione reductase, they catalyze the following reaction:

$$2GSH+ROOH\overset{Glutathione\;Peroxidase}{\to }GSSG+ROH+H_{2}O\;GSSG+NADPH\overset{Glutathione\;Reductase}{\to }2GSH+NADP$$

or

$$2GSH+H_{2}O_{2}\overset{Glutathione\;Peroxidase}{\to }GSSG+2H_{2}O\;GSSG+\beta -NADPH\overset{Glutathione\;Reductase}{\to }2GSH+\beta -NADP$$

in which GSH = glutathione, its reduced form; GSSG = glutathione, its oxidized form; β-NADPH = nicotinamide adenine dinucleotide phosphate, its reduced form; and β-NADP = nicotinamide adenine dinucleotide phosphate, its oxidized form.

The serum level of GPx is regarded as an indicator of the body’s antioxidant capacity. Low levels of this antioxidant may be linked to OS induced by obesity. The determination of GPx levels can be conducted spectrophotometrically by measuring the light absorption of the product resulting from the enzymatic reaction. This involves analyzing the enzymatic activity in the supernatant from cell culture, urine, serum, plasma, platelets, other biological fluids, and tissue extracts. Moreover, GPx activity can also be measured using a colorimetric assay kit for plasma, erythrocyte lysates, tissue homogenates, and cell lysates. The kit directly quantifies NADPH consumption in coupled enzymatic reactions. The color change in the biological sample treated with specific reagents is proportional to the enzymatic activity level in the sample and is assessed using a photometer at a wavelength of 340 nm [226,227,228]. 

(3) Glutathione reductase (GR). This is an enzyme that facilitates the conversion of oxidized glutathione (GSSG) back to its reduced, free form (GSH), thereby ensuring adequate levels of cellular GSH. Its activity as a biomarker can be assessed either spectrophotometrically in the UV range by tracking the consumption of NADPH, observing a decrease in absorbance at 340 nm, or using a colorimetric assay that monitors the increase in absorbance at 412 nm due to the reduction in 5,5′-dithio-bis-(2-nitrobenzoic acid) (DTNB) to 5-thio-(2-nitrobenzoic acid) (TNB) by GSH [229,230,231,232,233,234].

$$NADPH+H^{+}+GSSG\overset{Glutathione\;Reductase}{\to }NADP+2GSH\;GSH+DTNB\to GS-TNB+TNB$$

(4) Glutathione S-transferase (GST). Glutathione S-transferase (GST) encompasses a group of metabolic isoenzymes that exhibit antioxidant and detoxification activity. It plays a crucial role in the elimination of various xenobiotics from the body by facilitating the conjugation of the thiol group of glutathione with electrophilic xenobiotics. Assessing GST activity serves as a diagnostic method for OS and gauges the efficiency of detoxification mechanisms mediated by this enzyme. This activity can be measured using a colorimetric GST assay kit, which allows for the quantification of total GST activity in cellular and bacterial lysates, plasma lysates, erythrocytes, and tissue homogenates. The method relies on a GST-catalyzed reaction where the thiol groups of reduced glutathione are conjugated with 1-chloro-2,4-dinitrobenzene (CDNB).

$$GSH+CDNB\overset{Glutathione\;S-transferase}{\to }GS-DNB\;conjugate+HCl$$

The reaction product absorbs at 340 nm, and the rate of increase in absorption is directly proportional to the GST activity in the biological sample. The detection limit of the assay ranges from 10 to 300 μg/mL. Additionally, the Sanger Sequencing method can be employed. While Sanger Sequencing is a DNA or RNA sequencing technique and not directly used to measure GST enzymatic activity, it can be applied to determine the genetic sequence of the gene encoding the GST enzyme [235,236,237,238,239,240,241,242,243,244]. 

(5) Superoxide dismutase (SOD) and catalase (CAT). These are endogenous antioxidants. Superoxide dismutase is an enzyme that, together with catalase, converts the superoxide anion into molecular oxygen and water.

$$2{O\cdot }^{-}+2H^{+}\overset{Superoxide\;dismutase}{\to }H_{2}O_{2}+O_{2}\;H_{2}O_{2}\overset{Catalase}{\to }H_{2}O+\frac{1}{2}O_{2}$$

A reduction in the activity of these enzymes indicates decreased antioxidant activity in the body, which may be attributable to obesity, among other factors. The enzymatic activity of all three types (Cu/Zn, Mn, and Fe) of SOD can be measured from urine, serum, plasma, and other biological fluids, tissue extracts, cell lysates, and cell culture mediums, using colorimetric or spectrophotometric methods. Given that SOD catalyzes the conversion of superoxide into molecular oxygen and hydrogen peroxide, SOD activity can be determined by measuring the decrease in superoxide anion concentration. Colorimetric or spectrophotometric kits contain specific reagents that, in interaction with superoxide anions, produce an analytical signal related to SOD enzymatic activity, namely a colored compound detectable at a wavelength of 450 nm. The decrease in color signal is proportional to SOD’s inhibitory activity [245,246,247,248,249,250,251,252,253,254,255,256,257,258,259,260,261]. 

Colorimetric or fluorometric assays for assessing catalase activity are based on the principle that catalase is induced to catalyze the decomposition of a specific amount of hydrogen peroxide into water and molecular oxygen, after which the enzymatic reaction is halted by adding sodium azide, which inhibits catalase activity. The undecomposed hydrogen peroxide then reacts with a chromogenic reagent based on substituted phenol, which oxidatively couples with 4-aminoantipyrine in the presence of hydrogen peroxide and peroxidase. The resulting red quinoneimine dye can be measured colorimetrically at 570 nm or fluorometrically with excitation at 535 nm and emission at 587 nm. The activity of catalase in the sample is inversely proportional to the signal because the greater the catalase activity, the lesser the amount of undecomposed hydrogen peroxide, leading to lower absorption or fluorescence. The detection limit is in the pico-unit range for 1 micro-unit of catalase activity. Measurements can be performed in erythrocytes, whole blood or plasma, lysates of Gram-positive bacteria, homogenates of Gram-negative bacteria, and supernatants of food samples homogenized with buffer solution, using appropriate dilutions. The spectrophotometric method for determining catalase is useful only in the absence of or at very low concentrations of substances, such as proteins, that absorb in the UV radiation range at similar wavelengths and can interfere with the measurement of catalase absorption. When possible, interfering substances are removed, or a control sample without catalase is prepared for comparative measurements. A kinetic monitoring program spectrophotometrically tracks the concentration of peroxide at 240 nm. As the catalase-mediated decomposition reaction of the peroxide progresses, the amount of peroxide and consequently, the absorbance decreases. The rate of decrease, or the kinetic speed, is directly proportional to the catalase activity [262,263,264,265,266,267,268,269,270,271,272,273]. 

The reference values that are generally accepted for plasma vitamin C, plasma vitamin E, erythrocyte GPx, erythrocyte GR, serum GST, SOD, and CAT in children are included in Table 2.

(6) The ratio between the reduced form and the oxidized form of glutathione. Measuring the GSH/GSSG ratio in cells is frequently utilized to assess OS, as glutathione plays a critical role in protecting cells against OS [281,282]. 

(7) Elevated levels of protein and lipid oxidation. The presence and high concentrations of oxidation reaction products of proteins and lipids, such as malondialdehyde and carbonylated proteins, can serve as indicators of OS levels. Accordingly, increased levels of malondialdehyde have been observed in the adipose tissue of obese individuals [283,284,285,286,287]. 

(8) Reactive oxygen species (ROS). In the context of obesity, adipocytes within fat deposits can produce an excess of free radicals, contributing to increased OS. Although free radicals are challenging to quantify due to their reactivity and chemical instability, if they interact with biological molecules (such as proteins, lipids in cell membranes, etc.), they can alter their structure, leaving a unique “fingerprint”, or a specific chemical change (modified proteins, lipid oxidation products, etc.). This alteration can be detected and quantified analytically, resulting in biomarkers that can be used to assess the level of OS in the body or the effects of antioxidants. One such detectable biomarker of OS associated with obesity is the superoxide anion [288,289,290,291,292,293].

(9) Selenium. Selenium is a key component in the body’s defense mechanism against OS, as it forms an integral part of antioxidant enzymes known as selenoproteins. Thus, maintaining optimal selenium levels is crucial for the effectiveness of the antioxidant system. The assessment of selenium levels in the blood (serum, plasma, and whole blood) or tissues (hair and nails) serves as a measure of the body’s ability to withstand OS. A significant decrease in selenium levels may suggest compromised antioxidant functionality and is potentially linked to an increased risk of diseases associated with OS. There are numerous techniques for measuring selenium status in blood, urine, tissue, or other biological samples, including atomic absorption and emission spectroscopy, spectrometry, spectrophotometry, fluorescence spectrometry (spectrofluorimetry), potentiometric methods (direct potentiometry and potentiometric titration), and neutron activation analysis, among others [294,295,296]. Among these methods is Atomic Absorption Spectrometry (AAS) in various forms, such as Hydride Generation Atomic Absorption Spectrometry (HG-AAS) or Graphite Furnace Atomic Absorption Spectrometry (GFAAS), Flame Atomic Absorption Spectrometry (FAAS) Electrothermal Atomic Absorption Spectrometry (ETAAS) [297,298], High-Resolution Continuum Source Graphite Furnace Atomic Absorption Spectrometry (HR-CS GFAAS) [299], etc. A number of adjunct methods for processing biological samples have been developed for the separation/preconcentration of selenium species from complex matrices before their determination using AAS [300]. More recently, Inductively Coupled Plasma Mass Spectrometry (ICP-MS) has been refined [301,302,303,304] and it also features a broad range of variants, such as Dynamic Reaction Cell-Inductively Coupled Plasma-Mass Spectrometry (DRC-ICP-MS) [305]. For the analysis of selenium traces and their speciation, a series of combined methods have been refined, such as High-Performance Liquid Chromatography Hyphenated to Atomic Spectrometry or Mass Spectrometry (HPLC-HGAAS, HPLC-HGAAF or HPLC-MS) [306].

Selenium is a crucial component of various enzymatic systems, playing a significant physiological role. As observed, its key function as an antioxidant is closely linked to the maintenance of cellular health and the immune system, contributing to the overall functioning of an organism. In optimal doses, selenium acts as a beneficial antioxidant and helps balance many body functions. However, beyond certain thresholds, it can become extremely toxic. The accurate determination and monitoring of selenium levels in various biological samples, such as blood, plasma, or urine, are particularly important in this context. Thus, assessing selenium status in the body can be associated, on one hand, with the development and progression of significant pathologies, and on the other hand, with the degree of toxicity induced by its presence above certain levels. However, toxicity levels of selenium are not well defined and the diagnosis is mostly ascertained based on the presence of features of selenosis [307]. 

The nutritional status of a nutrient refers to its intake, retention, and metabolism. Accordingly, the selenium status in the body encompasses selenium intake, its presence in tissues/retention in the body, selenium excretion, and its function, with specific Se-biomarkers corresponding to each of these components. Selenium intake refers to the amount of this micronutrient obtained from the daily diet, which can vary significantly based on consumed foods. Of note, higher levels of selenium intake have been listed by various agencies to circumvent selenium toxicity. The levels proposed by the United States Food and Drug Administration (FDA) for children and adolescents are presented in Table 3 [307].

Nutritional status can be estimated using dietary journals or food frequency questionnaires, utilizing food composition tables, as well as dedicated applications and software. However, assessing selenium intake through these procedures involves a degree of approximation since the tools used are relative. For instance, food composition tables do not account for variations in soil selenium content by geographic area. Similarly, utilized software cannot integrate parameters such as variations in food selenium content, food preparation, and processing that may affect selenium content, as well as the specificity of living organisms, genetic factors, and health status that can influence the capacity to absorb and utilize ingested selenium. For this reason, assessing selenium intake through the mentioned methods should be correlated with information provided by other biomarkers analyzed in the laboratory. For example, plasma selenium levels can be used to gauge selenium intake, especially when the dietary intake is predominantly in the form of selenomethionine (SeMet), one of the most readily assimilated and utilized forms of exogenous selenium by the body [166,308,309].

The presence of selenium in tissues relates to the amount of selenium the body utilizes and stores, determined by measuring its concentration in various tissues or biological fluids, such as nails, hair, whole blood, erythrocytes, and plasma. Although the concentration of selenium in serum and plasma quickly reflects short-term exposure to this micronutrient and varies rapidly based on recent dietary intake, this biomarker is one of the most commonly used in assessing the body’s selenium status. Erythrocytic selenium provides an assessment of selenium status over a longer period, given the 120-day half-life of red blood cells in circulation. Selenium concentrations in nails and hair are considered superior biomarkers of selenium status because these tissues grow slowly and accumulate or retain selenium, providing stability to these parameters. Thus, biomarkers in nails and hair allow for an integrated evaluation and more accurate monitoring of long-term selenium exposure (even up to 10–12 months), while blood biomarkers indicate a much shorter-term, specific exposure. Selenium content in nails and hair is directly associated with the content of selenoproteins and organic forms of selenium in the body, especially SELENOP and SeCys, and is inversely related to the content of inorganic forms, such as selenite and selenate. Selenium concentrations in hair and nails serve more as biosensors for selenium excretion, alongside the determination of total selenium content in urine and feces excreted over several days [166,308,309]. 

Renal excretion is a major pathway for the elimination of selenium from the body (50–60% of the total), making the measurement of selenium concentration in urine a sensitive biomarker for short-term selenium intake. However, the volume of fluids consumed and urine excreted, as well as kidney health, significantly influence this parameter. For this reason, it is recommended to couple the evaluation of urinary selenium concentration with the determination of urinary creatinine concentration to correct for errors due to changes in urine volume based on fluid intake. The presence of selenium in tissues, or its retention in the body, can be determined from the difference between the exogenously admitted amount (diet and supplements) and the total selenium content in urine and feces. Since selenium is involved in numerous essential physiological functions, especially in defending the body against OS by incorporating it into a series of antioxidant enzymes, functional biomarkers refer to measuring the activity of these enzymes. Thus, assessing the activity of selenoprotein enzymes such as GPX1, GPX2, SELENOP, etc., allows for the evaluation of selenium availability for various biological functions. The relationship between the activity of selenoprotein enzymes and the body’s selenium status is quite direct. A selenium deficiency results in reduced activity of antioxidant enzymes, while normal or increased activity can indicate an adequate intake or, at very high values, a toxic selenium intake. For example, until reaching the plateau levels of selenoprotein P, its concentrations in circulating blood serve as a relatively accurate functional biomarker of selenium intake. However, when selenium concentrations increase significantly and exceed the saturation level of SELENOP, selenium toxicity, or selenosis, may be suspected due to surpassing toxic thresholds. In this way, circulating SELENOP also becomes a suitable biomarker for therapies with inorganic selenium that exceed the recommended intake [166,308,309,310,311].

### 3.5. Selenium Status and Obesity in Children and Adolescents

As previously stated, the BMI is an estimative parameter of obesity, especially in children and adolescents, because it does not distinguish between fat and muscle mass and does not provide information about the distribution of fat. Other procedures for assessing body composition, such as measuring waist circumference and imaging techniques, are not yet well mastered since the limits expressing pathology have not been clearly defined. Therefore, the search continues for biological or chemical indicators that can serve as biomarkers for assessing a person’s obesity status and type. In this context, among the markers that can contribute to portraying this pathology and its associated complications are blood glucose levels (an increased glycemia can be associated with obesity), the insulin/insulin-like growth factor ratio (INS/IGF-1 outside optimal parameters can indicate insulin resistance, often associated with obesity, the lipid profile (unbalanced values of LDL, HDL, and triglycerides are often found in obesity and associated pathologies), and the C-reactive protein ([CRP] as a marker of low-grade chronic inflammation that occurs in obesity). Furthermore, the assessment of leptin levels, which are elevated in obesity, and, respectively, adiponectin levels, which are lowered in the case of insulin resistance, can also contribute to completing this picture [312]. The integration of so-called “multi-omics” technologies, which encompasses multiple biological disciplines involved in evaluating the profiles of genes (genomics, epigenomics, or transcriptomics), proteins (proteomics), or small molecule metabolites (metabolomics) from cells or tissues, offers the possibility of identifying genetic and epigenetic biomarkers that can provide precise information about the etiology of obesity, its prevention, risk reduction, and the prevention of associated secondary diseases, as well as the development of rigorous and personalized treatments [313,314,315,316,317]. 

Selenium, an essential trace element with significant roles in human health, has been explored as a biomarker for obesity in children and adolescents. However, there is a great variability in the reference values of selenium in healthy children based on geographic area, age, and gender. Table 4 illustrates some reference values of serum selenium in children as cited in the literature.

The physiopathology of selenium status in relation to obesity in children involves intricate interactions between nutrient metabolism, OS, inflammatory responses, and endocrine regulation [130]. The relationship between selenium and obesity, especially in pediatric populations, is complex, influenced by both genetic and environmental factors [325,326,327].

In the setting of obesity, the build-up of excess adipose tissue significantly escalates OS, leading to persistent activation of inflammatory pathways and insulin resistance [328,329]. It can also affect thyroid function in children, including alterations in thyroid hormone levels and metabolism, thereby exacerbating metabolic imbalances. Triiodothyronine (T3) plays a direct role in regulating the expression of LDL receptor (LDL-R) and apolipoprotein B (apoB). Additionally, it is established that a significant portion of T3 is produced from thyroxine (T4) in peripheral tissues through the process of 5′-deiodination dependent of a selenoprotein, whose activity diminishes in cases of selenium deficiency [330,331,332,333,334,335].

Obesity is marked by chronic low-grade inflammation, fueled by an increase in pro-inflammatory cytokines and adipokines produced by the expanded adipose tissue [336]. Selenium plays a pivotal role in modulating inflammatory processes through its regulation of the nuclear factor kappa-light-chain-enhancer of activated B cells (NF-κB), a crucial transcription factor in the gene expression of inflammatory markers. Selenoproteins, such as selenoprotein S and GPx, are key in modulating the inflammatory response [141,143,337,338,339]. Therefore, optimal selenium levels are crucial to mitigate OS and its detrimental effects in obese children [326,330,339,340,341].

A study carried out by Mahmoud KG et al. investigates the role of selenoprotein-P as a biomarker for selenium status in obese children and adolescents, concluding that levels were significantly lower in these categories. This research highlights the significance of selenium and its carrier proteins in the nutritional evaluation of obese children, suggesting the potential value of selenium status, especially via selenoprotein-P levels, as a biomarker for obesity in the pediatric population [342].

The link between selenium status and obesity does not exist in isolation but is influenced by a host of dietary and lifestyle factors. For instance, the consumption of a high-calorie, nutrient-poor diet can contribute to both obesity and suboptimal selenium intake. Physical activity, in turn, can affect selenium status through its effects on body composition and inflammation. Thus, the interplay between selenium, diet, and lifestyle choices is a critical area of consideration in the physiopathology of childhood obesity [175,343,344].

In children, obesity is a growing global concern, with significant implications for physical and mental health. Investigating selenium status in obese children is crucial because nutritional deficiencies or excesses during childhood can have profound effects on growth, development, and long-term health. Some studies suggest that obese children may have an altered selenium status, which could influence the development of obesity-related complications such as insulin resistance, hypertension, and dyslipidemia. However, the evidence is mixed, and the direction of the relationship remains unclear [175,326,330,341,344,345,346].

Emerging research underscores the intricate relationship between trace elements, metabolic health, and obesity in the pediatric population, drawing on diverse studies that highlight the role of selenium among other minerals. One study elucidates the connection between urinary metals, including selenium, and fasting blood glucose levels in children, further investigating how physical activity can alter these associations, suggesting a complex link between trace elements and metabolic health in young individuals [347]. Concurrently, research focusing on selenium’s bio-accessibility post-digestion emphasizes the importance of age in assessing selenium bioavailability and its implications for obesity and metabolic health, underscoring the necessity of age-specific dietary guidelines [348]. Investigating data from the NHANES database, this study delves into the relationship between mineral consumption, such as selenium, and obesity rates in children, offering crucial insights into the impact of mineral intake on the risk and handling of obesity within pediatric groups. It reveals that children with obesity tend to have higher dietary intakes of selenium [349]. Additionally, a study on children and adolescents with obesity reveals decreased blood levels of essential elements involved in antioxidant defense and metabolic control, including selenium, pointing to OS and micronutrient imbalance as significant factors in the pathophysiology of obesity among youth [350].

Exploring the connection between selenium status and obesity in adolescents involves delving into the intricate interplay between trace mineral nutrition, metabolic health, and the unique physiological and environmental challenges faced during adolescence. This period of life is characterized by rapid growth, hormonal changes, and often, the adoption of lifestyle habits that can affect health well into adulthood [351,352]. Research on selenium status and obesity in adolescents has yielded mixed results, with some studies indicating altered selenium levels in obese individuals compared to their lean counterparts, and others finding no significant differences [353,354]. These discrepancies highlight the complexity of selenium metabolism and its interaction with obesity-related factors such as diet quality, physical activity levels, and genetic predisposition.

The dietary sources of selenium—primarily seafood, meat, eggs, and certain nuts and seeds—may vary widely in availability and consumption patterns, further complicating the assessment of selenium’s role in child and adolescent obesity. Additionally, the potential for selenium to both ameliorate and exacerbate aspects of metabolic health suggests a need for careful consideration of selenium status in dietary and clinical interventions aimed at managing obesity [355].

## 4. Conclusions

In the 21st century, obesity is undoubtedly a “complex multifactorial” medical condition that has reached epidemic proportions globally. In the WHO European region, excess weight and obesity represent the fourth most common risk factor for the development of non-communicable diseases, after hypertension, malnutrition, and tobacco use.

Obesity in children and adolescents can have major negative effects on their health and impair the proper functioning of organ systems in the body (cardiovascular, endocrine, reproductive, digestive, immune, respiratory, skeletal, muscular, excretory, nervous, etc.) with serious short- or longer-term consequences.

Oxidative stress represents an imbalance between the production of free radicals and the body’s ability to neutralize or detoxify these free radicals.

Oxidative stress and obesity are in a bidirectional connection; they influence and reinforce each other, having a significant impact on health and can lead to the development of some of the most serious chronic conditions.

Oxidative stress may play an important role in the development of obesity through several mechanisms including damage to cells/cell components and tissues in the body, especially to adipocytes and mitochondria, leading to excessive fat accumulation and reduced fat burning, causing chronic inflammation, which can affect carbohydrate and lipid metabolism and cause insulin resistance and influence hormone levels in the body, including hormones that regulate hunger and satiety (ghrelin, leptin, cholecystokinin, neuropeptide y, etc.).

Obesity can increase and be a source of oxidative stress because it is associated with chronic inflammation. Fat cells produce bioactive substances called adipokines that can induce an excess of free radicals and can affect carbohydrate and lipid metabolism and lead to insulin resistance, which can increase the production of ROS.

Selenium, through its incorporation into selenoproteins such as GPx, TrxR, and others, plays a crucial role in neutralizing excess free radicals and protecting cells against oxidative stress. This function is vital for maintaining cellular redox homeostasis, regulating signal transduction, cell proliferation, and aging, and protecting against various forms of cellular damage that can lead to diseases including cancer, cardiovascular diseases, and neurodegenerative disorders. The biochemical advantage of selenium over sulfur in selenocysteine, such as faster kinetics in oxidation–reduction reactions and better reversibility, underscores its essential role in cellular antioxidant defense mechanisms and metabolic processes.

The multifaceted actions of selenium and selenoproteins extend beyond antioxidant defense to include significant roles in DNA synthesis and repair, thyroid hormone metabolism, immune system enhancement, and reproductive health. Furthermore, selenium nanoparticles present a promising avenue in biomedicine, offering enhanced bioavailability, lower toxicity, and improved therapeutic efficacy compared to other selenium forms. This indicates selenium’s potential in a wide range of applications, from combating oxidative stress and inflammation to preventing and treating serious diseases such as cancer, cardiovascular diseases, and infections. Extensive research supports the critical balance of selenium in the body, highlighting the detrimental effects of both deficiency and excess and pointing to its necessity for overall health and disease prevention.

The robust interplay between OS and obesity is well documented, revealing a reciprocal relationship that significantly impacts health outcomes. Oxidative stress, characterized by an overproduction of ROS due to diminished antioxidant defenses, is both a precursor and a consequence of obesity. This cycle suggests a pressing need for comprehensive studies to unravel the mechanisms at play, which would pave the way for developing more effective treatments for obesity and its complications. Biomarkers such as vitamin C, vitamin E, GPx, and selenium have been identified, indicating the body’s compromised ability to combat oxidative stress in obesity. These biomarkers, ranging from exogenous antioxidants to endogenous enzymes, highlight the crucial role of diet and antioxidant intake in managing oxidative stress levels. Moreover, the role of selenium is underscored not just as a part of the body’s defense against oxidative stress but also as a critical indicator of overall health, given its involvement in a broad spectrum of physiological functions. The balance of selenium intake, retention, and metabolism emerges as a vital aspect of health, reflecting the complex interactions between diet, oxidative stress, and obesity. This synthesis underscores the importance of a holistic approach to obesity management, integrating dietary modifications and targeted interventions to boost the body’s antioxidant defense and address the multifaceted challenges presented by OS and obesity.

The relationship between selenium status and obesity in children is characterized by a complex web of interactions involving antioxidant defense, thyroid hormone metabolism, and inflammatory processes. While the precise mechanisms and directionality of these relationships continue to be explored, it is evident that maintaining an adequate selenium status could play a role in mitigating some of the adverse metabolic effects associated with obesity in pediatric populations. Future research should aim to elucidate the optimal selenium levels for supporting metabolic health in children, taking into account individual variability and the broader nutritional context.

Selenium plays a complex role, both as a nutrient and potential biomarker, in the context of pediatric obesity. Its interactions with metabolic processes, dietary intake, and bio-accessibility are crucial factors in understanding and managing obesity in children and adolescents.

Given the equivocal nature of the relationship between selenium and obesity in children, more research is needed to clarify the underlying mechanisms. Understanding whether selenium status is a contributor to or a consequence of obesity could inform nutritional interventions and public health strategies aimed at preventing and managing obesity from an early age. Moreover, it is essential to consider the balance of selenium intake; both deficiency and excess have adverse health implications, emphasizing the need for precise nutritional recommendations tailored to individual needs.

## Figures and Tables

**Table 1:** BMI percentiles of weight status classification according to age and gender.

Weight Status	BMI Percentiles
underweight	<5th percentile
normal weight	5th percentile–85th percentile
overweight	85th percentile–95th percentile
obesity	>95th percentile
severe obesity	>99th percentile

**Table 2:** Reference values of biomarkers of OS in children.

Biomarker	Reference Value in Children	Reference
plasma Vitamin C	5.0–15.0 mg/L (28.4–85.2 µmol/L)	[274]
plasma Vitamin E	11.9–30.0 µmol/L	[275]
erythrocyte GPx	12.96–33.15 U/g Hb; 52.92 U/g Hb	[276,277,278,279]
erythrocyte GR	6.74–13.25 U/g Hb	[278,279]
serum GST	4.25–9.32 U/g Hb	[278]
SOD	1161.80 U/g Hb	[280]
CAT	51.92 × 10^4^ IU/g Hb	[280]

**Table 3:** US FDA upper levels of selenium dietary intake for children.

Age Range	Male/Female [µg/Day]
infancy	45–60
1–3 years	90
4–8 years	180
9–13 years	280
14–18 years	400

**Table 4:** Reference values of serum selenium in healthy children according to region and age.

Age Range	Country	Serum Se Reference Interval [μ/L]	Reference
0–2 months	US	45–90	[318]
3–6 months		50–120	
7–9 months		60–120	
10–12 months		70–130	
13 months–17 years		70–150	
0–1.5 years	UK	30.0–49.7	[319]
1.5–4 years		45.0–90.0	
5–16 years		55.3–115.3	
37–42 weeks	Different countries from EU, US, Canada, and Japan	20.5–69.5	[320]
<18 months		26.1–76.6	
18 months–3 years		40.3–88.4	
4–18 years		47.4–101.9	
1–16 years	Iran	63–106	[321]
5–14 years	New Zealand	73.5–79.0	[322]
2.1–6.6 years	Brazil	47–142	[323]
<1 month	Not applicable, review	15–107	[324]
1–2 months		15–100	
2–4 months		10–93	
4–12 months		13–116	
1–5 years		34–129	

## Data Availability

No new data were created or analyzed in this study. Data sharing is not applicable to this article.
